# Thermal Burns and Smoke Inhalation: A Simulation Session

**DOI:** 10.7759/cureus.360

**Published:** 2015-10-21

**Authors:** Michael Parsons, Justin Murphy, Sabrina Alani, Adam Dubrowski

**Affiliations:** 1 Emergency Medicine, Memorial University of Newfoundland; 2 Medicine, Memorial University of Newfoundland; 3 Emergency Medicine, Oncology, Memorial University of Newfoundland; 4 Emergency Medicine, Pediatrics, Memorial University of Newfoundland; 5 Marine Institute, Memorial University of Newfoundland

**Keywords:** trauma, simuation, carbon monoxide, burns, poisoning, cyanide

## Abstract

In recent years, simulation-based training has seen increased application in medical education. Emergency medicine simulation uses a variety of educational tools to facilitate trainee acquisition of knowledge and skills in order to help achieve curriculum objectives. In this report, we describe the use of a highly realistic human patient simulator to instruct emergency medicine senior residency trainees on the management of a burn patient.

## Introduction

Burns are commonly classified as thermal, electrical, or chemical, with thermal burns further subdivided as secondary to flame, contact, or scalding [1]. Thermal burns affect thousands of people every year, and it is estimated that approximately 500,000 individuals require treatment, while around 10% require hospital admission [2]. Associated carbon monoxide (CO) and cyanide (CN^-^) toxicity from smoke inhalation can increase morbidity and mortality and should be treated accordingly [3-4]. Advances in emergency care, including wound care, fluid resuscitation, and timely consultation, have led to an improved prognosis for burn victims [1-2]. For emergency medicine trainees, knowledge and practical experience in dealing with burn emergencies is essential.

This technical report describes a simulation-based teaching session designed for a cohort of postgraduate emergency medicine trainees in their third and final year of training at Memorial University of Newfoundland. The objective of this case study is to educate trainees about significant burns and underlying associated conditions, such as CO toxicity, CN toxicity, and trauma.

**Figure 1:**
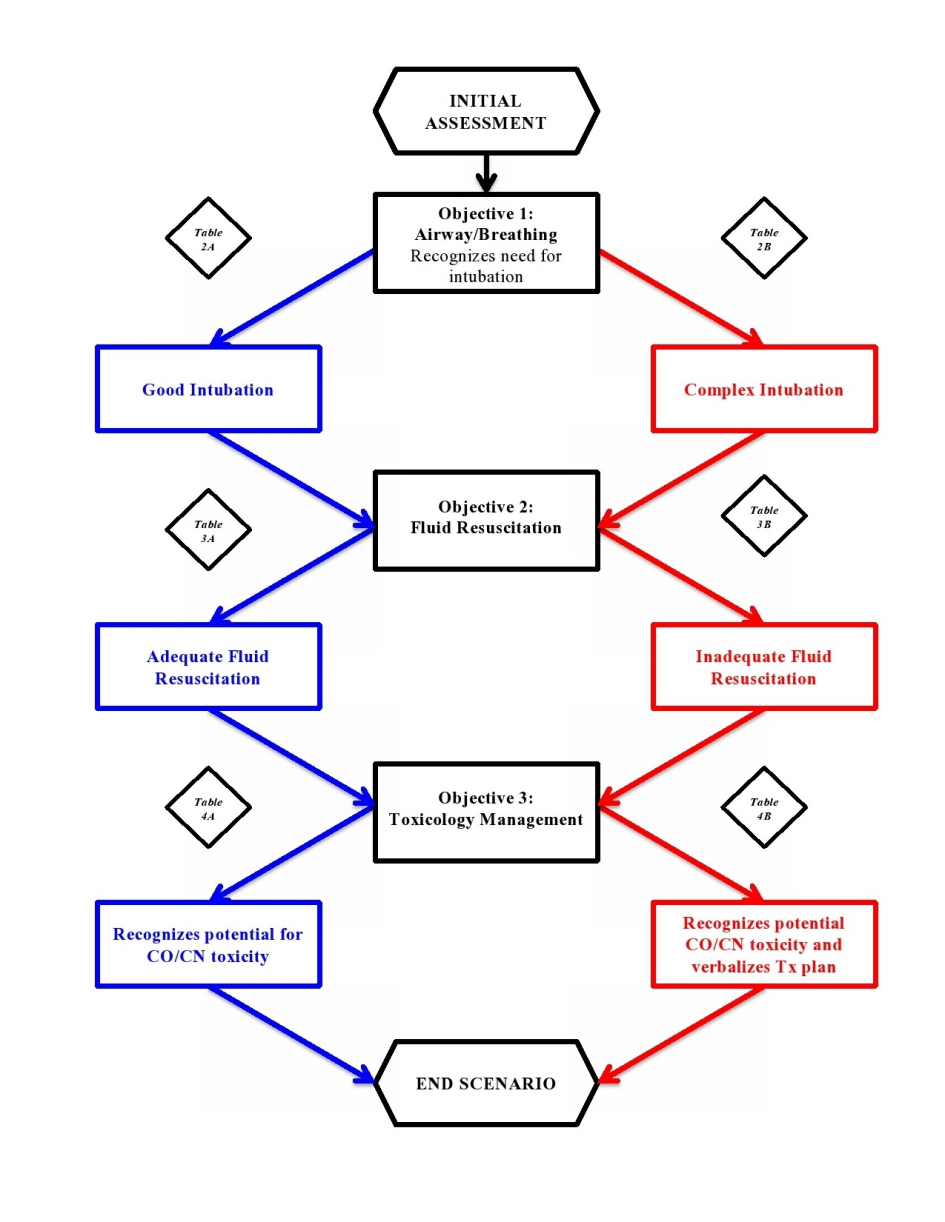
Flow Chart Outlining The Steps To Follow For Burn Case Simulation

In this scenario, we use a highly realistic and technologically-advanced human patient simulator operated by a trained technician who follows a pre-defined storyboard (i.e. simulation scenario). The difficulty of the scenario can be tailored to the level of the trainee with modifications to one or more objectives and final case outcomes/endpoints for the scenario. Figure 1 provides a general overview of key objectives, decision points and flow of the case.

## Technical report

The simulation training session is conducted in a simulation laboratory, using our Laerdal SimMan 3G human patient simulator. Prior to the session, a detailed stepwise scenario template is developed. The simulation technical staff prepare the human patient simulator and the laboratory space for execution of the case. At our site, early submission of cases to the simulation laboratory staff technologist (generally six weeks ahead) is followed by a 'dry run' of the case. This allows all personnel involved to become familiar with the case and to address any practical concerns. A trained confederate plays the role of the ER nurse. When running the case for learners, there is a minimum of two instructors/clinicians present. One individual sits with the laboratory staff technician in the control room and directs the flow of the case while a second observes the scenario and takes notes on resident performance. Based on the principles of the optimal challenge point framework [5-6], we developed a progressively challenging case that can be adjusted in the level of difficulty for learners at different levels of training (Tables 1-2). Previous research suggests that such progressive adjustments in difficulty may be more beneficial to the learning process [7-8]. Figure 1 outlines the basic flow of the scenario and references Tables 2-4 to give direction with respect to varying levels of difficulty and key potential 'turning points' in the case.

**Table 1:** A Stepwise, Detailed Scenario Template

Pre Scenario		
A 55 year old male arrives to the ED via EMS. A fire was noted by an upstairs tenant. They attempted to enter the basement apartment but were unsuccessful. EMS called. Fire rescue arrived first. Patient removed from house. CPR required for 2 minutes. At ER is drowsy but has regained Blood Pressure, pulse and respiratory effort.		
History		
Little available from EMS (limited information from bystanders/neighbour). Look for medic alert, wallet, and old records/ pharmacy.		
Allergies	?	
Medications	?	
PMHx	?	
Other	HR 130, BP 100/60, Temp 37.6, RR 26, Gluc 10, Sat 93% 100%O2	
HEENT	Nasal singeing, soot around and in mouth	
Neurological	Extraocular movements intact, PERRL, GCS-8	
Cardiovascular	Tachycardic, Low Blood Pressure	
Respiratory	Wheeze diffusely, spontaneous respirations, RR22	
Abdomen	Soft; 50% abdominal wall 2nd degree burn	
Extremities	Anterior thighs and forearms burned- 2nd degree	
Expected Actions		
Rapid initial ABCDE assessment with brief AMPLE history		
C-Spine precautions		
Calculate %BSA burn		
Place patient on telemetry		
Obtain IV access		
Administer oxygen		
Obtain ECG		
IV Fluids		
Order labs – electrolytes, BUN, creatinine, complete blood count, liver function panel, arterial blood gas, serum lactate, blood/urine cultures, urinalysis, coagulation panels, cardiac troponin, amylase, T&S, CO & CN levels		
Objective 1: Airway/Breathing		
Stage	Vitals	Expected Actions
Pt's respiratory status starts to deteriorate	HR 130, BP 100/60, temp 37.6, RR 26, Gluc 10, Sat 93% 100%O2	Identifies extensive burn and likely airway involvement and need for intubation. Anesthesia assistance should be considered but is not available. Obtain airway cart and prepare for intubation.
Intubation	HR 130, BP 90/60, Sats 93% on 100%O2, RR vent	Difficult, but the use of bougie and/ or Glidescope allows successful intubation. Adequate sedation is needed, but administration of meds (Propofol, morphine, midazolam, fentanyl) leads to slight worsening of BP. Also need for aggressive fluid resuscitation, with consideration of airway burn and drawbacks of too much fluid
Paralysis	HR 135, BP 80/50, Sats 85-90% on 100% O2	Results in failed airway attempts and need for surgical airway. Same sedation and BP considerations as above. Same fluid resuscitation considerations as above.
Results from ordered labs	ABG- sat 93% on 100%, pO2 150, pCO2 60, pH 7.15 Lactate- 3.5 CBC- Leukocytes 17, HGB 140 Lytes, BUN, Creatinine - nil	Proceed with fluid resuscitation and further management.
Objective 2: Fluid Resuscitation		
Adequate fluid resuscitation	HR 120-130, BP 90-100 systolic, RR vent, sats 90-93% on 100% O2	Adequate resuscitation requires appropriate use of Parkland Formula. Adequate fluid resus allows patient to stabilize, but further sedation is needed. Analgesia should be considered. Trainee may also consider the use of vasopressors to support blood pressure.
Inadequate fluid resuscitation	HR 135, BP 80-90/60, temp unchanged, RR vent	Intubated patient remains hypotensive and is also not adequately sedated. Reassess ABC's, and reassess to attempt appropriate fluid resuscitation.
Objective 3: Toxicology and Management		
Normal toxicology	HR 120, BP 90-100 syst, RR vent, sats 90-93% on 100%	Trainee should verbalize concerns about CO and CN and have ordered/ followed up on labs to investigate these. Normal values do not require consultation with hyperbarics, but Trauma surgery team needs to be consulted otherwise patient's labs will begin to deteriorate. Patient needs to be transported to ICU for monitoring and wound management.
Elevated CO/CN levels	CO levels 30%	Trainee should verbalize concerns about CO and CN and have ordered/ followed up on labs to investigate these. Elevated results require hyperbarics and trauma surgeon to be consulted. Patient transported to ICU for further management.

**Table 2:** Turning points and expected actions for the Airway/Breathing section

2A - Good Intubation		
Stage	Vitals	Expected Actions
Recognize the extensive burn and the signs of airway involvement.	HR 130 BP 100/60 RR 32 Sats 93% on 100% O2	Trainee should call for the airway cart and prepare for intubation Anesthesia backup can be considered but is not available. Trainee prepares for a difficult airway, verbalizes the P's of Rapid Sequence Intubation (Preparation, Preoxygenation, Pretreatment, Paralysis, Positioning, Placement) and considers awake intubation.
Intubation		Ensures adequate sedation Recognizes caution with use of paralytics Administer meds (eg. Ketamine, midazolam, fentanyl) Use of bougie to assist intubation If steps done appropriately- intubation goes well
Post intubation vitals: Sedation leads to further hypotension	HR 135 BP 80/60 RR- ventilated Sat 90-93% on 100% O2	Recognizes need for aggressive fluid resuscitation
Lab Results: ABG - Sats 93% on 100%, pO2 150, pCO2 60, pH 7.15, Lactate 3.5 CBC - Leukocytes 17, HGB 140, Lytes, BUN, Creatinine - nil *Proceed with fluid resuscitation and further management.*		
2B – Complex Intubation		
Stage	Vitals	Expected Actions
Recognize the extensive burn and the signs of airway involvement.	HR 130 BP 100/60 RR 32 Sats 93% on 100% O2	Trainee should call for the airway cart and prepare for intubation Anesthesia backup can be considered but is not available. Trainee prepares for a difficult airway, reviews the 6Ps and considers awake intubation.
Preparing for Intubation, vitals worsening	HR 135 BP 90/60 Sats dropping	Ensures adequate sedation Administer meds (eg. Ketamine, midazolam, fentanyl)
To surgical airway pathway: Chosen case difficulty- simulated airway edema/ swelling Learner blindly gives paralytics		Recognize difficult airway and specific issues/ need for surgical airway Proceed to surgical airway
Surgical airway		See Appendix 1 for side table set-up of low- fidelity surgical airway model [15].
Post-intubation vitals: Sedation meds lead to further hypotension	HR 135 BP 80/60 RR- ventilated Sats 90-93% on 100%	Recognizes need for aggressive fluid resuscitation
Lab Results: ABG - Sats 93% on 100%, pO2 150, pCO2 60, pH 7.15, Lactate 3.5 CBC - Leukocytes 17, HGB 140, Lytes, BUN, Creatinine - nil *Proceed with fluid resuscitation and further management.*		

**Table 3:** Turning points and expected actions for the Fluid Resuscitation section

3A – Adequate Fluid Resuscitation		
Stage	Vitals	Expected Actions
Post-intubation, hypotension	HR 135 BP 80/60 RR- ventilated Sats 90-93% on 100% O2	Trainee recognizes need for fluid resuscitation Verbalizes use of Parkland Formula as starting point Foley catheter to monitor output
Adequate fluid resuscitation allows patient to stabilize, but is still inadequately sedated.	HR, BP transient response with fluids Ongoing need for sedation / analgesia and associated challenges with BP	Provides analgesia and sedation. Continues aggressive IV fluids Consider vasopressors for blood pressure support.
Fluid resuscitation addressed	HR 120 BP 90-100 systolic RR- ventilated	
Persistent difficulty oxygenating	Sat 90-93% 100% O2	Trainee verbalizes that O2 Sats are still not increasing and suggests the need for toxicology screening to rule out CO and/or CN poisoning
3B – Inadequate Fluid Resuscitation		
Stage	Vitals	Expected Actions
Post-intubation, hypotension	HR 135 BP 80/60 RR- ventilated Sat 90-93% on 100% O2	Trainee attempts fluid resuscitation Does not verbalize or follow the Parkland Formula. Inadequate volume
Patient remains hypotensive and inadequately sedated.	HR 140 BP 70/30 RR- ventilated Sat 90-93% 100% O2	Prompts: Nurse states “his HR is going up, do you want to do anything with that?” or “should we put in a foley?”
If learner addresses fluids, the BP will improve, but the patient is still inadequately sedated.	HR, BP transient response with fluids Ongoing need for sedation / analgesia and associated challenges with BP	Provides analgesia and sedation. Continues aggressive IV fluids Consider vasopressors for blood pressure support.
Fluid resus addressed	HR 120 BP 90-100 systolic RR- ventilated	
Persistent difficulty oxygenating	Sat 90-93% 100% O2	Trainee verbalizes that O2 Sats are still not increasing and suggests the need for toxicology screening to rule out CO and/or CN poisoning

**Table 4:** Turning points and expected actions for Toxicology Management section

4A – Junior Learner- Toxicology & End Scenario		
Stage	Vitals	Expected Actions
Recognizes potential TOX	HR 120 BP 90-100 systolic RR- ventilated Sats 90-93% on 100% O2	Verbalize concerns about CO and CN toxicity. Trainee orders labs to investigate.
Junior learner	END SCENARIO	Continues monitoring, reassessment, meds for sedation and analgesia Consult Trauma team Consult ICU Provides succinct “SBAR” (Situation, Background, Assessment, Recommendation) type case summary
4B – Advanced/ Senior Learner- Toxicology & End Scenario		
Stage	Vitals	Expected Actions
Recognizes potential TOX	HR 120 BP 90-100 systolic RR- ventilated Sats 90-93% on 100% O2	Verbalize concerns about CO and CN toxicity. Trainee orders appropriate investigations
Senior learner	END SCENARIO	Continued monitoring, reassessment, meds for sedation and analgesia Consult hyperbarics Consider CN kit Consult Trauma team Consult ICU Provides “SBAR” (Situation, Background, Assessment, Recommendation) type case summary Begin plans for transport, in consultation with specialty services, if in a community hospital setting

### Pre-briefing

A pre-briefing is held with the trainees before details of the case are presented. The pre-brief plays a very important role in the case and for simulation-based medical education as a whole. Every effort is made to establish a 'safe container' for the learners, as described by Rudolph, et al. [9]. All those involved in observing the case, including the sim lab staff who will manage the technical aspects of the case, will be disclosed to the residents. We inform the residents of whether or not the case will have an evaluative function but emphasize our focus on learning. Limitations of the simulation are acknowledged, specifically, any technical issues with the mannequin and resource availability. Without revealing specific details, the origin and rationale for the case may be discussed to help the learner appreciate why the specific case has been included in the curriculum.

### Case

Once the pre-brief is finished, the brief background, case history, and vitals are presented to the learner/team in the pre-brief room, as outlined under the pre-scenario information in Table 1. Residents are then instructed to move to the simulation room and begin the case with an assessment of the patient. 

The scenario takes place in the resuscitation bay of a community hospital. It involves a middle-aged male patient arriving at the emergency department via Emergency Medical Services (EMS). The patient was rescued from a house fire and presents with extensive burns and evidence of smoke inhalation. When requested, trainees are provided with a very limited history lacking details on allergies, medications, and past medical history (PMHx).

**Figure 2:**
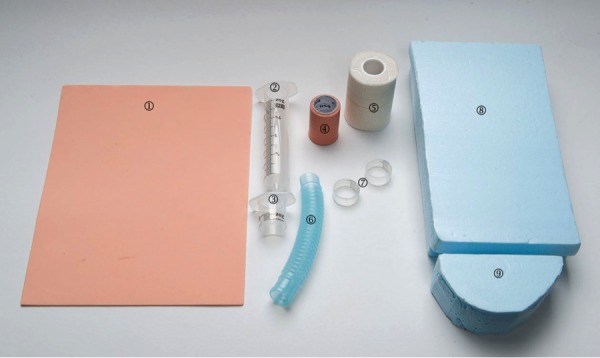
Cricothyroidotomy – All materials Materials include: 1. Craft sponge (Multicraft Imports material in “flesh”) 2. 60 cc syringe 3. 60 cc syringe: cut 2 inches at proximal end 4. Leukoplast Sleek ® (BSN Medical) 5. Tensoplast ® (BSN Medical) 6. Corrugated Tubing 7. 60 cc syringe: cut 1.5 cm from central portion of syringe 8. Styrofoam block: cut piece X size 9. Styrofoam block: cut piece X size *Not pictured: Blue Duct Tape

**Figure 3:**
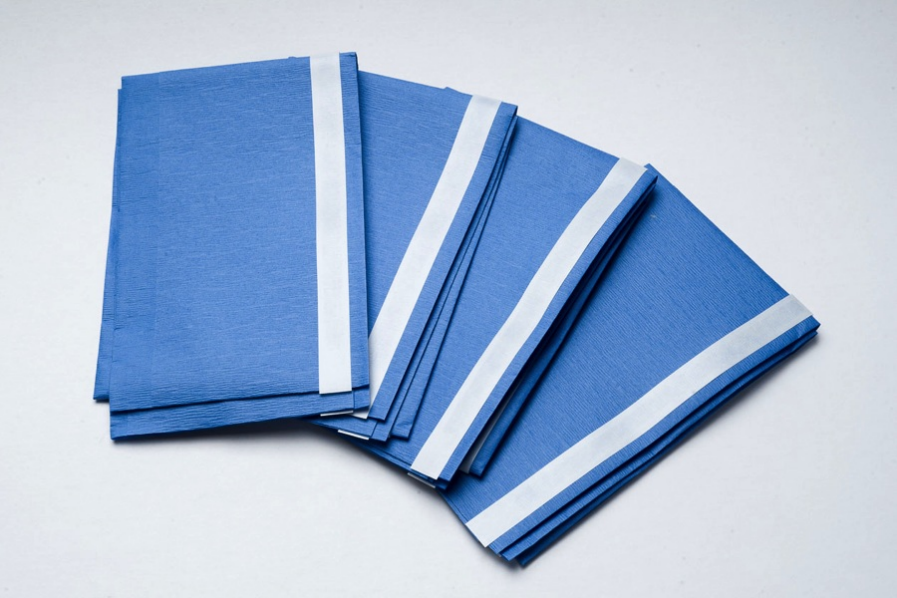
Cricothyrotomy – Drape Towels Used to drape adequately in order for appropriate exposure to conduct surgical airway skills (surgical Cricothyrotomy) *N.B. green draping (or any reusable draping) can be substituted

**Figure 4:**
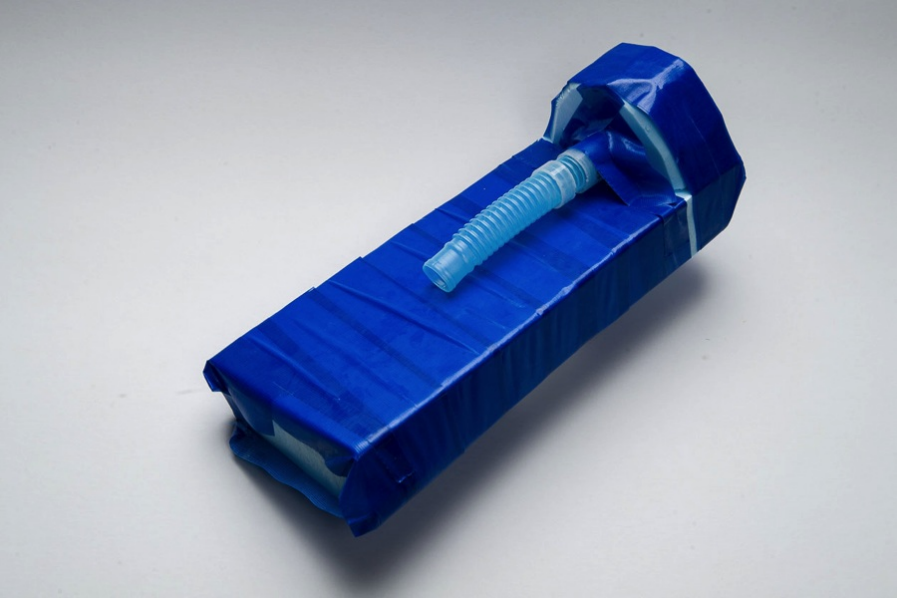
Cricothyrotomy – Simulated Neck/Chin/Thyroid & cricoid Cartilage, Simulated Trachea 1. Simulated neck and chin: place 9 at a 90° angle to 8 at the top of 8 and secure with duct tape 2. Simulated Thyroid cartilage: place 3 perpendicular and in the center of 9 and secure with duct tape 3. Simulated trachea: Insert 6 securely into 3 4. Slide 7 onto 6 leaving approximately 2-3 ridges between the end of the simulated thyroid cartilage (3) and where 7 rests

**Figure 5:**
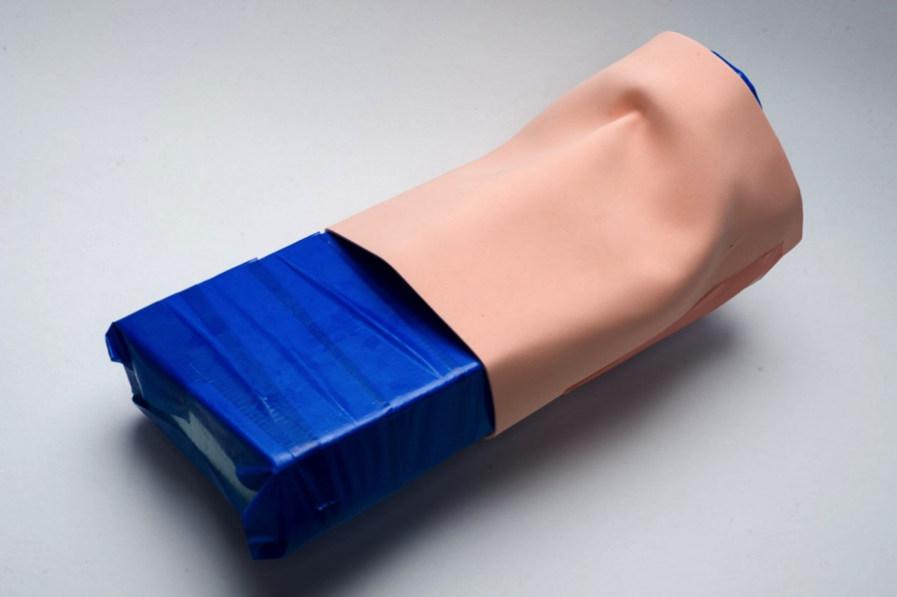
Cricothyrotomy – Simulated Skin Attached to Trachea 1. Place 1 over 8 and 9, should cover from chin to past the lower end of the corrugated tubing (6) 2. Fashion in such a manner as to simulate a neck 3. Use 5 to help fashion neck by manipulating around the upper part of the corrugated tubing (6) and under the simulated chin (9) 4. Secure with adhesive (5) at edges

**Figure 6:**
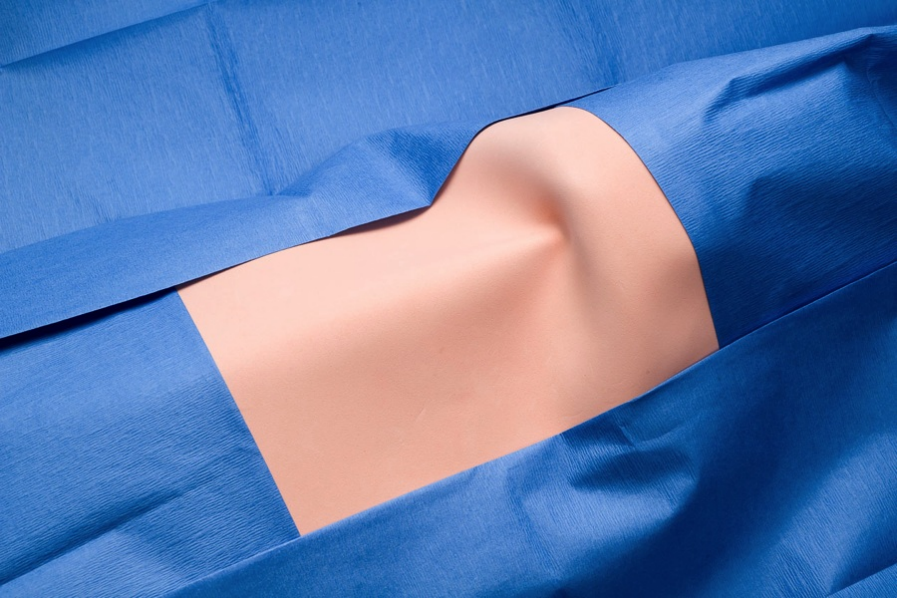
Cricothyrotomy – Final Product (Draped Close-Up) Simulated neck draped with adequate exposure to perform surgical airway skills (surgical Cricothyrotomy) This model is *reusable*; the simulated skin attached (1) and corrugated tubing (6) should be replaced between users. *N.B. For a more refined model, hot wire cutters can be used to accurately cut Styrofoam and shape to best emulate a chin. Additionally, an alternate attachment for the simulated thyroid cartilage (tracheal syringe piece 3) could be achieved by bolting down this piece to 9 rather than securing with adhesive (duct tape).

The scenario begins with the patient in the emergency resuscitation room. At the learner's request, the patient will be connected to the monitor and have intravenous (IV) lines established. Available equipment includes a resuscitation cart, defibrillator, and difficult airway equipment. Drugs necessary for pain management, advanced life cardiac support, and rapid sequence intubation, as well as props for fluid resuscitation and simple wound care are available, if requested by the trainee. In addition, if a surgical airway is required, learners can practice their cricothyroidotomy skills on a low fidelity surgical airway model as outlined in Figures 2-6.

### Debriefing

A structured debriefing session is carried out after completion of the scenario. Care is taken during the debriefing to ensure that the debriefer to resident ratio approximates 1:1. This limit encourages trainees to speak more freely about challenges, thought processes, and issues they may have faced during the simulation. Our debriefing model is based mainly on frame-discovery as described by Rudolph, et al. [10]. This approach focuses on an advocacy-inquiry technique aimed at uncovering the trainee’s thought processes, allowing us to address both errors of process and knowledge gaps.

### Post-scenario didactics

A didactic session is routinely integrated into the debriefing session. This enables the instructors to address knowledge gaps identified through the scenario and debriefs and gives trainees an opportunity to solidify and consolidate knowledge gained as a result of the simulation exercise. Instructors are prepared to discuss a couple of specific topics in detail as a part of the debriefing process, but we recognize the need for flexibility and will modify the main discussion topics if particular issues arise during the simulation session.

## Discussion

The ability to diagnose and treat thermal burns, smoke inhalation, and associated toxicology is crucial for the practicing emergency physician. For a number of reasons, many emergency medicine trainees gain limited hands-on exposure of managing burn patients in an emergency clinical setting. A case-based simulation of this clinical presentation can prove invaluable. Key learning objectives include:

1.  Recognizing and managing extensive thermal burns with airway involvement - including surgical airway management,

2. Recognizing and addressing challenges of fluid management in the burn patient,

3. Recognizing potential toxic exposures in the burn patient,

4. Integrating and discussing key relevant non-medical expert CanMEDs roles, with a particular focus on crisis resource management.

The post-scenario didactic session allows for discussion on how to emergently care for a burn victim with extensive thermal injuries and a high likelihood of airway burns, smoke inhalation, and secondary toxicological issues. The didactic portion of the session draws upon several key resources available to the emergency physician [2-3]. The discussion will include the preferred method of intubation and the challenges of the emergent surgical airway. Appropriate fluid resuscitation, based on the Parkland formula, is reviewed and bedside intervention for potential CO/CN exposure is addressed [2-3]. Topics in crisis resource management (CRM) also fit very well with the case. In a setting where the learner has limited resources and is faced with a sick patient who potentially needs a bedside surgical airway and certainly requires subsequent specialist assistance, CRM is easily integrated as a key objective and may affect long-term learning and performance [11-14].

In this technical report, our use of a stepwise approach to facilitate the execution of a simulation-based scenario gives a structured but flexible template that accounts for potential variation in resident approach to the case. The dry run helps to ensure the case runs smoothly and helps identify practical issues with using the scenario. The use of a formal debriefing model combined with a post-scenario didactic session allows instructors to identify and address knowledge gaps and errors of process encountered with their trainees.

## Conclusions

Teaching emergency medicine trainees how to care for burn victims and to treat injuries secondary to smoke inhalation through simulation-based medical training (SMBE) is a valuable training tool. Here, we described a stepwise algorithm developed to facilitate the execution of a simulation scenario as well as an integrated teaching session incorporating a range of simulators and didactics with components of debriefing included.
